# Propulsion Phase Characteristics of Loaded Jump Variations in Resistance-Trained Women

**DOI:** 10.3390/sports11020044

**Published:** 2023-02-09

**Authors:** Timothy J. Suchomel, Shana M. McKeever, Olusegun Sijuwade, Logan Carpenter

**Affiliations:** 1 Department of Human Movement Sciences, Carroll University, Waukesha, WI 53186, USA; 2 Divine Savior Holy Angels High School, Milwaukee, WI 53222, USA; 3 Aurora Baycare Sports Medicine Center, Green Bay, WI 54311, USA

**Keywords:** jump squat, hexagonal barbell jump, trap bar jump, jump shrug, force–velocity profile

## Abstract

The purpose of this study was to compare the propulsion phase characteristics of the jump squat (JS), hexagonal barbell jump (HEXJ), and jump shrug (JShrug) performed across a spectrum of relative loads. Thirteen resistance-trained women (18–23 years old) performed JS, HEXJ, and JShrug repetitions at body mass (BM) or with 20, 40, 60, 80, or 100% BM during three separate testing sessions. Propulsion mean force (MF), duration (Dur), peak power output (PP), force at PP (F_PP_), and velocity at PP (V_PP_) were compared between exercises and loads using a series of 3 × 6 repeated measures ANOVA and Hedge’s *g* effect sizes. There were no significant differences in MF or Dur between exercises. While load-averaged HEXJ and JShrug PP were significantly greater than the JS, there were no significant differences between exercises at any individual load. The JShrug produced significantly greater F_PP_ than the JS and HEXJ at loads ranging from BM–60% BM, but not at 80 or 100% BM. Load-averaged V_PP_ produced during the JS and HEXJ was significantly greater than the JShrug; however, there were no significant differences between exercises at any individual load. Practically meaningful differences between exercises indicated that the JShrug produced greater magnitudes of force during shorter durations compared to the JS and HEXJ at light loads (BM–40%). The JS and HEXJ may be classified as more velocity-dominant exercises while the JShrug may be more force-dominant. Thus, it is important to consider the context in which each exercise is prescribed for resistance-trained women to provide an effective training stimulus.

## 1. Introduction

Jumping exercises are commonly used to enhance the force production characteristics of an individual during the triple extension of the hip, knee, and ankle (plantar flexion) joints. This is likely due to their technique simplicity, specificity to common movements in sports (e.g., jumping, sprinting, and change of direction), and ballistic intent, which avoids undesirable deceleration at the end of the propulsion phase of the movement [1,2]. In fact, researchers have shown that heavier loads can increase force production during jumping [3], indicating that loaded jumps may provide an effective way to progress the training intensity of jumping exercises [4]. While there are a variety of loaded jump possibilities such as jumps performed with barbells, dumbbells, chains, elastic bands, etc., limited research has compared the force production characteristics between jump variations [5,6]. Therefore, further insight on the differences between loaded jump types may help strength and conditioning practitioners to implement these exercises more effectively with their athletes.

Swinton et al. [5] indicated that the hexagonal barbell jump (HEXJ) produced a significantly greater jump height, peak force, peak power output (PP), and rate of force development compared to the jump squat (JS). The authors concluded that the placement of the external load at arm’s length rather than the upper back contributed to a greater mechanical advantage and, ultimately, a superior performance. This rationale is supported by additional researchers who showed greater force, velocity, and power output during HEX deadlifts compared to straight barbell deadlifts [7,8]. Another study by Suchomel and colleagues [6] compared the power production characteristics of the JS, HEXJ, and jump shrug (JShrug) using loads based on percentages of body mass (BM). Their results indicated that the HEXJ and JShrug produced greater PP compared to the JS; however, each exercise possessed unique force and velocity characteristics. For example, the JShrug produced the greatest force at peak power (F_PP_) while the HEXJ produced the greatest velocity at peak power (V_PP_). While the previous studies provide some insight into the force production characteristics of the JS, HEXJ, and JShrug, no research has compared these exercises with female participants. Thus, to best serve both men and women who may use these exercises in training, further research is warranted.

The training stimulus within a loaded jump may vary based on the external load that is prescribed. Much of this research has focused on identifying the load that optimizes PP. For example, researchers have shown PP may occur at loads as low as the individual’s BM (i.e., unloaded) [9,10] or between loads ranging from 30–60% of the individual’s one repetition maximum (1RM) back squat [11]. Additional researchers examining the HEXJ concluded that PP occurred with loads ranging from 20–40% of a 1RM back squat [5] or 10–20% of a 1RM box squat [12]. Compared to the other two exercises, the JShrug is typically loaded using a percentage of a 1RM weightlifting catching derivative [13,14,15,16,17,18,19,20,21,22,23]. In this light, PP has been shown to be maximized between 30–45% of a 1RM hang power clean [14,16,18,20]. However, Suchomel and colleagues [6] indicated that PP was also maximized between 20–40% BM, which the authors determined to be approximately 15–26% of a 1RM back squat. Despite these findings, none of these studies investigated the loading effects within a female population. Thus, because of the impact that load placement and loading can have on propulsive performance characteristics, it is important that strength and conditioning practitioners understand the differences between commonly prescribed exercises within both male and female populations. Therefore, the purpose of this study was to compare the propulsion phase characteristics of the JS, HEXJ, and JShrug performed across a spectrum of loads. It was hypothesized that the JShrug would produce greater force production characteristics compared to the JS and HEXJ at lighter loads, but these differences would become much smaller at heavier loads. In addition, it was hypothesized that the JS and HEXJ would produce greater velocity characteristics compared to the JShrug across all loads and that the differences would become larger at heavier loads.

## 2. Materials and Methods

### 2.1. Design

The current study used a repeated measures design to identify the differences in power production characteristics between the JS, HEXJ, and JShrug performed with only the participants’ BM and with 20, 40, 60, 80, and 100% of the participants’ BM [24,25]. Each participant attended a familiarization session and three separate testing sessions over two weeks.

### 2.2. Participants

Thirteen resistance-trained women (age: 21.5 ± 3.2 years (18–23 years), height: 165.6 ± 5.3 cm, BM: 66.1 ± 5.9 kg, relative back squat 1RM: 1.4 ± 0.2 kg·kg^−1^) who have lifted a minimum of twice per week for the past two years participated in this study. While each participant had previous experience with the JS and HEXJ exercises, this was not the case with the JShrug. However, it should be noted that each participant was familiar with and had previously trained with the power clean and its derivatives. The participants each attended four separate testing sessions that included a 1RM back squat and jump familiarization session and three separate exercise testing sessions. The participants were recruited via word-of-mouth. Exclusion criteria included a current or previous musculoskeletal injury that prevented participation (e.g., muscle or joint injury) as identified by an orthopedic professional, and unfamiliarity with loaded jumps. The study was conducted according to the guidelines of the Declaration of Helsinki and was approved by the Institutional Review Board of Carroll University (#17-032; approved 30 October 2017). Each participant read and signed a written informed consent form prior to participation.

### 2.3. 1RM Back Squat and Jump Familiarization

During the 1RM back squat and jump familiarization session, the principal investigator collected the age, BM, height, and estimated 1RM back squat of each participant. The participants then completed a standardized warm-up that including three minutes of light-moderate stationary cycling and dynamic stretching (e.g., lunges, straight leg march, walking quadriceps stretch, etc.) [6]. Following the warm-up, each participant completed a 1RM back squat protocol using previously described methods [26]. A 1RM back squat was completed to provide further information about the training status of the participants and to relate it back to the loads that optimized their power development. Prior to maximal squat attempts, the participants completed a self-selected barbell warm-up before performing back squat sets: five repetitions at 30%, five repetitions at 50%, three repetitions at 70%, and one repetition at 90% of their estimated 1RM. Following the warm-up sets, the principal investigator and research assistants adjusted the load for each of the 1RM attempts using a minimum increase of 2.5 kg. Two minutes of rest were provided between the first two warm-up sets while a minimum of three and maximum of five minutes were provided between the last two warm-up sets and 1RM attempts. A successful 1RM squat attempt required the participants to squat to a depth in which the top of their thigh at a minimum was parallel to the ground. This was visually monitored by the principal investigator and the research assistants.

After the 1RM squat was determined, the participants were provided with a self-selected rest period before being familiarized with the JS, HEXJ, and JShrug technique and the individual exercise testing protocol in the following sessions. The principal investigator and research assistants demonstrated and discussed the jumping cues used during the testing sessions before the participant practiced each exercise using a submaximal load. Because several participants had not performed the JShrug exercise, they were coached through the movement using previously discussed cues [27].

### 2.4. Jump Testing Sessions

As mentioned above, the participants attended an additional three separate testing sessions following the 1RM sessions to perform sets of either the JS, HEXJ, or JShrug. The first session took place one week after the 1RM and familiarization session and the remaining sessions were separated by 48–72 h with the order of exercises being randomized. At the start of each session, the BM of the participant was recorded to determine the loads for each exercise and to allow for ratio scaling of performance variables. The participants performed the same standardized warm-up described above and warm-up repetitions with 30 and 50% of their BM. After completing the warm-up, the participants performed two maximal effort repetitions of either the JS, HEXJ, or JShrug with 0, 20, 40, 60, 80, and 100% BM. It should be noted that the 0% condition was performed with a PVC pipe weighing less than one kilogram while other loads were performed with various barbells (7.5, 15, and 20 kg) and bumper plates. Lifting straps were used to prevent grip strength from being a limiting factor for the HEXJ and JShrug during repetitions performed with 60% BM or higher. The load order was randomized prior to each participant’s first exercise testing session and remained consistent throughout the remaining sessions. This was done to help prevent a fatigue or potentiation order effect. Finally, one minute of rest was provided between the testing repetitions while two minutes were given between loads.

All JS, HEXJ, and JShrug repetitions were performed on a mounted, in-ground force platform Model 6090-06 (Bertec Corporation, Columbus, OH, USA) sampling at 1000 Hz. Briefly, each participant stood motionless for one second with the external load prior to receiving a countdown of “3, 2, 1, Jump!” Each exercise was performed based on previous descriptions [6,27] whereby the participants performed a countermovement prior to jumping as high as possible. The primary differences between the exercises were the placement of the load and how the countermovement was performed based on the position of the external load. Briefly, the JS was performed while the load was held on the upper back, like a back squat. The HEXJ was performed with the participant standing within the HEX PVC frame or barbell and holding the external load at arm’s length. Finally, the JShrug required participants to begin the movement in the mid-thigh (power position) [28]. Thus, the JS and HEXJ allowed the participant to perform a countermovement almost entirely in the vertical plane, while the JShrug required participants to perform a hip hinge movement [6].

### 2.5. Data Analyses

Raw force-time data collected from the force platform were exported into a customized spreadsheet (Microsoft Inc., Redmond, WA, USA) for analysis. System weight (BM + external load) was determined while the participants stood motionless on the force platform prior to initiating the countermovement [29]. A threshold of five times the standard deviation of the first second of the quiet standing phase was to determine the onset of each jump type. Using this method, the onset of each jump was determined to have occurred 30 milliseconds prior to the instant where vertical force decreased below the calculated threshold [29]. The force-time data were then integrated to calculate the velocity-time data while the power–time data were then calculated as the product of force and velocity at each time point. The propulsion phase of each jump was at the point when system velocity first exceeded 0.01 m·s^−1^ following the onset of the jump and ended when force dropped below system mass [30]. Propulsion mean force (MF), duration (Dur), peak power output (PP), force at peak power (F_PP_), and velocity at peak power (V_PP_) were calculated for comparison. The greatest magnitude of power output was identified as peak power output and the corresponding force and velocity produced at this point were identified as force at PP (F_PP_) and velocity at PP (V_PP_), respectively. Relative MF, F_PP_, and PP were calculated by dividing each magnitude by the participant’s BM. Finally, time-normalized force–time curves of each exercise performed at each load were produced using previously outlined procedures [23]. Using this method, we resampled force–time data to 500 data points in which each data point corresponded to 0.2% of the total movement time.

### 2.6. Statistical Analyses

The Shapiro–Wilks test was used to assess the normality of each variable while the relative and absolute reliability were assessed using two-way mixed methods intraclass correlation coefficients (ICC) and typical error expressed as coefficients of variation percentages (CV%), respectively. The ICC and CV% magnitudes were interpreted based on previous recommendations [31,32]. Three (exercise) × six (load) repeated measures ANOVA with Bonferroni *post hoc* tests were used to compare the propulsive characteristics between the JS, HEXJ, and JShrug exercises and between loads (i.e., 0, 20, 40, 60, 80, and 100% BM). Greenhouse–Geisser adjusted values were used if the assumption of sphericity was violated. Practical differences between exercises and loads were calculated using Hedge’s *g* effect sizes. Effect sizes magnitudes were interpreted as trivial, small, moderate, large, very large, and nearly perfect if Hedge’s *g* values equaled 0.00–0.19, 0.20–0.59, 0.60–1.19, 1.20–1.99, 2.00–3.99, and ≥4.00, respectively [33]. The average of each variable and time-normalized force–time curves was used for statistical analyses. All statistical analyses were performed within SPSS 28 (IBM, Chicago, IL, USA) and the criterion level for statistical significance was set at *p* ≤ 0.05. Data are presented as mean ± standard deviation.

## 3. Results

Descriptive data for the JS, HEXJ, and JShrug are displayed in Table 1. Each of the variables was normally distributed. All data for the JS (ICC = 0.92–0.99, CV = 1.2–9.2%), HEXJ (ICC = 0.92–0.99, CV = 1.4–7.1%) were highly reliable and showed acceptable variability. While the JShrug showed good–excellent relative reliability (ICC = 0.72–0.99) for all variables and acceptable variability for propulsion mean force, PP, F_PP_, and V_PP_ (CV = 2–7.6%), propulsion duration variability ranged from 2.8–12.3% with unacceptable variability at 60 (12.3%) and 100% (11.4%) BM.

### 3.1. Exercise Main Effect

Statistically significant exercise main effects existed for PP (*p* = 0.004), F_PP_ (*p* < 0.001), and V_PP_ (*p* < 0.001), but not for MF (*p* = 0.323) or Dur (*p* = 0.438). Post hoc analysis indicated that the HEXJ (*p* = 0.002) and JShrug (*p* = 0.044) produced significantly greater PP compared to the JS. JShrug F_PP_ was significantly greater than the JS (*p* = 0.001) and HEXJ (*p* = 0.013). In contrast, JS (*p* = 0.043) and HEXJ (*p* < 0.001) V_PP_ were significantly greater than the JShrug while the HEXJ V_PP_ was also greater than the JS (*p* = 0.002).

### 3.2. Load Main Effect

**Figure 1:**
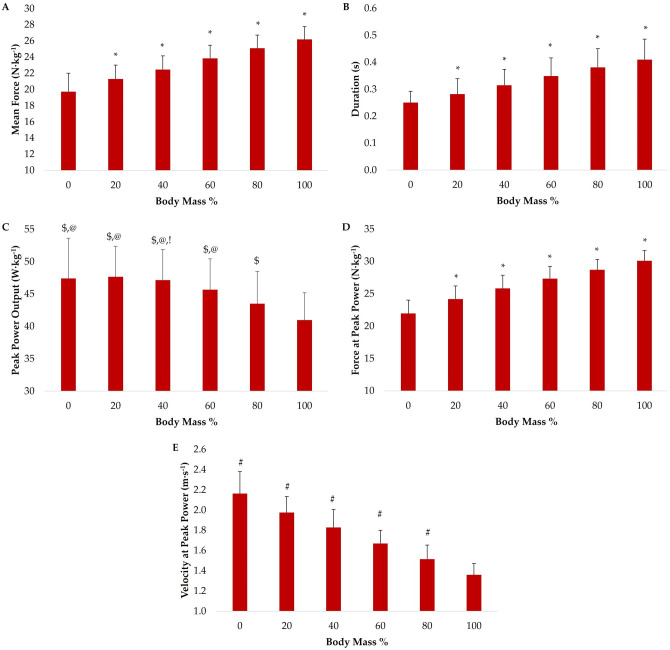
Load main effect post hoc comparisons for propulsion (**A**) mean force, (**B**) duration, (**C**) peak power output, (**D**) force at peak power, and (**E**) velocity at peak power. Data are presented as mean ± standard deviation. * = significantly greater than each lighter load (*p* < 0.01); $ = significantly greater than 100% BM (*p* < 0.01); @ = significantly greater than 80% BM (*p* < 0.01); ! = significantly greater than 60% BM (*p* < 0.05); # = significantly greater than each heavier load (*p* < 0.001).

Statistically significant load main effects existed for MF, Dur, PP, F_PP_, and V_PP_ (all *p* < 0.001). *Post hoc* analyses are displayed in Figure 1.

### 3.3. Exercise x Load Interaction Effect

**Figure 2:**
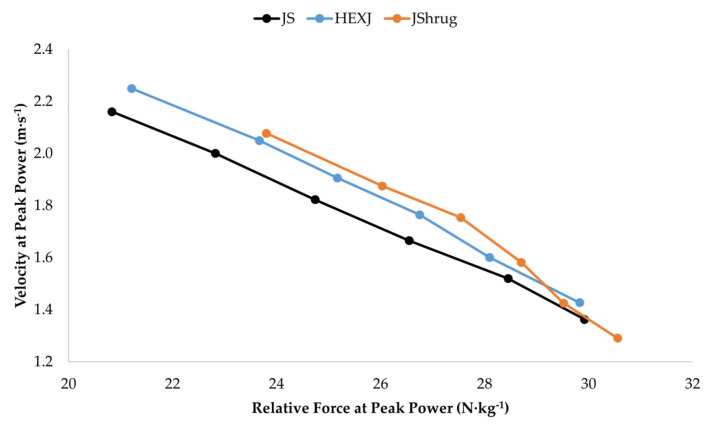
Jump squat (JS), hexagonal barbell jump (HEXJ), and jump shrug (JShrug) force-velocity curves performed at body mass and with 20, 40, 60, 80, and 100% of body mass.

Figure 1 and Figure 2 show the loading effects and force-velocity profiles of each exercise, respectively. Significant exercise × load interaction effects existed for PP and F_PP_ (both *p* < 0.001) but not V_PP_ (*p* = 0.423). Post hoc analysis indicated that the JShrug produced greater F_PP_ than both the JS and HEXJ at BM and with 20, 40, and 60% BM (all *p* < 0.01), but not 80% (*p* = 0.253 and *p* = 0.069) or 100% BM (*p* = 0.914 and *p* = 0.721). There were no differences in F_PP_ between the JS and HEXJ across all loads (*p* > 0.05). In addition, post hoc analysis indicated that there were no significant differences in PP between exercises at any load (*p* > 0.05). It should be noted that no MF or Dur interaction effects were reported due to a lack of statistically significant load main effects.

### 3.4. Force–Time Curve Comparisons

**Figure 3:**
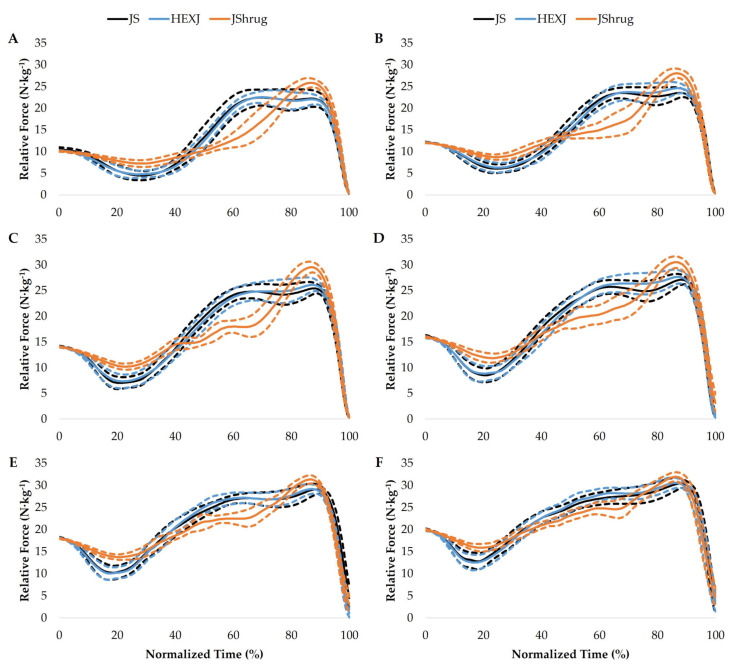
Jump squat (JS), hexagonal barbell jump (HEXJ), and jump shrug (JShrug) time-normalized relative force–time curves performed at body mass (**A**) and with 20 (**B**), 40 (**C**), 60 (**D**), 80 (**E**), and 100% (**F**) body mass. The solid lines represent the average participant relative force at each time point, while the dashed lines represent the 95% upper and lower confidence intervals.

The force–time curve of the JS, HEXJ, and JShrug performed at BM and with 20, 40, 60, 80, and 100% BM are displayed in Figure 3. The JS and HEXJ force–time curve confidence intervals overlapped throughout the entire movement at every load. In contrast, the JShrug displayed unique force–time characteristics at different portions of the total movement percentage compared to the JS and HEXJ. These were present during BM at 14.4–38.8%, 50.4–71.4%, and 83.2–92.6%; at 20% BM: 14–36.6%, 49.8–72%, 82.6–92%; at 40% BM: 9.2–34.2%, 45.6–71.2%, and 82.2–92%; at 60% BM: 8.6–29%, 44.6–68.6%, and 82–90.8%; at 80% BM: 6.6–25.6%, 49.2–68.2%, and 85.6–89.6%; and 3.6–21.8% at 100% BM.

## 4. Discussion

The current study examined the force production characteristics of the JS, HEXJ, and JShrug performed with different percentages of the participant’s BM. There were no main effect differences in propulsion MF or Dur between exercises. While greater PP was produced during the HEXJ and JShrug compared to the JS based on exercise main effects, there were no significant differences between exercises at any of the examined loads. The JShrug produced greater F_PP_ than both the JS and HEXJ at loads ranging from BM-60%, but not at 80 or 100% BM. While greater V_PP_ was produced during the JS and HEXJ compared to the JShrug based on exercise main effects, there were no significant differences between exercises at any load. As hypothesized, there was a decrease in velocity and an increase in force with an increase in load, across all exercises. Finally, the JShrug displayed unique force–time characteristics compared to the JS and HEXJ across all loads.

Jump performance is ultimately determined by the propulsion impulse produced by an individual [34]. Moreover, researchers have concluded that the force–time curve shape [35] and propulsion force [36] determine how the impulse was created. Given that propulsion impulse is the product of MF and Dur, the current study sought to provide insight on the underlying characteristics of each exercise. As expected, propulsion MF and Dur increased as the external load increased. Interestingly, there were no significant exercise main effect differences in propulsion MF or Dur when averaged across all loads. It should be noted however, that moderate effect sizes existed between the JShrug and the JS (*g* = 0.99 [0.18–1.81] and *g* = −1.15 [−1.99–−0.32]) and HEXJ (*g* = 0.71 [−0.08–1.51] and *g* = 1.05 [−1.87–−0.23]) for both MF and Dur, respectively, during the BM condition. Although the confidence intervals for the MF differences between the JShrug and HEXJ crossed zero, the other MF and Dur differences between the JShrug and other exercises should be considered practically meaningful. Simply, the participants produced greater force over a shorter duration during the propulsion phase of the JShrug, indicating that this exercise may promote the development of rapid force production characteristics to a greater extent compared to the JS and HEXJ using a PVC pipe or PVC hexagonal bar. While the JShrug maintained the shortest propulsion phase durations as the external load increased, none of these differences was practically meaningful while trivial–small effect sizes were present (*g* = −0.51–0.04). In contrast, JShrug MF was the highest with loads ranging from BM–60% with trivial–moderate effects being present depending on the load (*g* = 0.09–0.70), but then became the lowest at 80 and 100% BM. These findings support the conclusions of previous researchers that the JShrug provides an effective training stimulus at lighter loads [6,13,14,15,16,17,18,19,20]. However, due to the technique of the exercise, the stimulus may be significantly modified as loads continue to increase [6].

While previous researchers have shown that the HEXJ and JShrug produced significantly greater PP compared to the JS in resistance-trained men [6], the current findings contrast with these findings as there were no significant differences in PP between exercises at any load. It should however be noted that moderate effect sizes (*g* = 0.70–0.73) existed when comparing the JShrug and JS at loads ranging from BM–40% while only trivial–small effect sizes (*g* = 0.06–0.27) existed between the JShrug and HEXJ at the same loads. In common with previous studies [5,6], greater PP was produced during the HEXJ compared to the JS with small–moderate effect sizes existing across loads (*g* = 0.36–0.61). However, none of the previously discussed differences was practically meaningful. Interestingly, the women in the current study produced the greatest PP during the JShrug compared to the other exercises at BM and with 20 and 40% BM; however, the greatest PP was produced during the HEXJ for the remaining loads. This is likely due to large drop-offs in PP with heavier loads during the JShrug. Suchomel and colleagues [6] indicated that the JShrug had the greatest decrease in PP across loads compared to the JS and HEXJ (9.5 W·kg^−1^) in resistance-trained men. The women in the current study showed slightly larger decreases across the same loading spectrum (10.0 W·kg^−1^) while the JS and HEXJ only decreased 4.8 and 5.9 W·kg^−1^, respectively. Given that the greatest PP for the JS and HEXJ was produced at 20% BM and the JShrug at BM, this should not be surprising. In fact, the effect sizes displayed in Table 1 indicate that the differences between the load that maximized PP in each exercise and 100% BM were moderate–large and practically meaningful. This was also the case for the JShrug performed at 80% BM. Therefore, while the JS, HEXJ, and JShrug can all be implemented at lighter loads to maximize PP in resistance-trained women, it appears that the JShrug should be loaded with a slightly lower load. As noted in previous research [6], this is likely due to the mechanical differences between the exercises.

An interesting aspect of this study is the investigation of the F_PP_ and V_PP_ characteristics of the examined exercises. While it was unsurprising that force increased and velocity decreased with a greater external load, the current results suggest that the JShrug appears to be a more force-dominant exercise compared to the JS and HEXJ. This is supported by the practically meaningful effect sizes between the JShrug and JS from BM–60% (*g* = 1.39–2.16) and HEXJ from BM–80% BM (*g* = 0.89–1.62). These findings are in line with previous research in resistance-trained men [6]. While the JShrug produced the greatest F_PP_ across the examined loading spectrum, the force–velocity profile of the exercise (Figure 2) shows a clear drop-off in force and velocity with loads greater than 40% BM. In fact, only moderate differences existed in F_PP_ between the 60, 80, and 100% BM loads (Table 1). As mentioned above and in previous research [6,16,18], these findings are likely due to a breakdown in the JShrug technique at heavier loads. In contrast, the force–velocity profiles of the JS and HEXJ were relatively linear with more steady increases and decreases in F_PP_ and V_PP_ as the load increased, respectively. Interestingly, the HEXJ produced greater F_PP_ compared to the JS at loads ranging from BM–60%; the JS produced slightly greater force than the HEXJ at 80 and 100% BM. While all the differences between the JS and HEXJ were trivial–small, these findings partially support previous literature that has shown greater kinetic outputs (e.g., force, rate of force development, etc.) during HEX exercises compared to traditional barbell exercises [5,6,7,8].

The HEXJ produced the greatest V_PP_ at every load in the current study and was followed by the JS and JShrug. These findings are in line with previous research that has compared the HEXJ with other loaded jumps [5,6]. Despite a significant exercise main effect, there were no significant differences in V_PP_ between exercises at any of the examined loads. From an effect size standpoint however, the differences between the HEXJ and JS were small–moderate (*g* = 0.34–0.87) but were moderate–large (*g* = 0.80–1.60) when compared to the JShrug. In addition, the differences between the JS and JShrug were small–moderate (*g* = 0.37–0.82). Although these effect sizes were not practically meaningful, the consistent moderate–large effect sizes across loads and the force–velocity profiles of each exercise (Figure 2) allow us to conclude that the JS and HEXJ may be more velocity-dominant exercises compared to the JShrug. While the JShrug is considered one of the fastest weightlifting derivatives [37], it is important that strength and conditioning practitioners understand that there are other exercises that may provide a superior velocity stimulus. For clarity, this conclusion does not suggest that the JShrug cannot be used as a high-velocity stimulus; rather, it is important to consider the context in which it is prescribed for resistance-trained men and women.

Similar to the power–time curve analysis of previous research [6], the JShrug appears to possess unique force–time characteristics compared to the JS and HEXJ. These differences are likely due to the technique of each exercise. For example, while the JS and HEXJ countermovement phases are performed almost entirely in the vertical plane, the JShrug requires a hip hinge movement [27]. This is evident from the force–time curves of each exercise (Figure 3) where the JShrug displays a smaller reduction in force during the unweighting phase [19]. The other unique force–time characteristics were displayed during the early and late portions of the propulsion phase. There appeared to be a more rapid rise in force and greater peak force during the JShrug compared to the JS and HEXJ. This was particularly evident at the lighter loads (BM–40%) but diminished at heavier loads. It should be noted that the rapid force production characteristics of the JShrug may be underpinned by its hip hinge movement. For example, the hip hinge of the JShrug may allow individuals to recruit hamstring musculature that may contribute to rapid force production after shortening [14]. Combined with the transition back to the mid-thigh (power) position [38], often noted as the strongest position in weightlifting movements, this may allow individuals to develop large magnitudes of force rapidly. This notion is supported by greater MF and F_PP_ produced during the JShrug over a shorter Dur.

To the authors’ knowledge, this is only the second study to compare the performance of different loaded jumps using percentages of BM [6]. While this may be viewed as a limitation due to other studies using percentages of a 1RM squatting variation [5,12], there are currently no criteria to determine a 1RM JShrug. Furthermore, even if a 1RM for each exercise could be determined, it would be difficult to compare the performances of each exercise since the relative loads of each would differ from an absolute standpoint. Using BM as a loading method possesses several limitations (e.g., relative strength, body composition, etc.); however, the current findings may also be converted into percentages of a 1RM back squat if both forms of data are collected. For example, each of the current exercises would maximize PP between loads of 0–26% of their 1RM back squat. Another limitation of the current study was the lack of kinematic data collected. Since the magnitude of the load and its placement can modify an individual’s technique, future research may consider examining the differences between these exercises using 3D motion analysis. Finally, it should be noted that this study only included resistance-trained women aged 18–23 years. Thus, the findings of this study may only be applied to this specific population.

## 5. Conclusions

The JShrug may be classified as a more force-dominant movement compared to the JS and HEXJ. Moreover, the JShrug may provide resistance-trained women with an effective training stimulus for rapid force production using loads ranging from BM–40%. However, strength and conditioning practitioners should note that a considerable drop-off in PP will likely result at heavier loads. The JS and HEXJ may be classified as velocity-dominant exercises when compared to the JShrug; however, the HEXJ may provide the greatest training stimulus especially as loads increase. It is recommended that the JS, HEXJ, and JShrug are prescribed using lighter loads to maximize PP. In addition, strength and conditioning practitioners should consider the training phase in which these exercises are implemented to provide their athletes with the most effective training stimulus.

## Figures and Tables

**Table 1:** Propulsive phase characteristics of the jump squat, hexagonal barbell jump, and jump shrug performed at body mass (BM) and with 20, 40, 60, 80, and 100% of BM. Data are presented as mean ± standard deviation.

Load (% BM)	Variable				
	MF (N·kg^−1^)	Duration (s)	PP (W·kg^−1^)	F_PP_ (N·kg^−1^)	V_PP_ (m·s^−1^)
Jump Squat					
0	18.7 ± 2.6	0.26 ± 0.04	45.0 ± 5.4	20.8 ± 1.8	2.16 ± 0.22
*g*	*−3.28 (−4.46*–*−2.10)*	-	−0.13 (−0.90–0.64)	*−5.10 (−6.69*–*−3.52)*	-
20	20.7 ± 1.7	0.29 ± 0.50	45.6 ± 4.5	22.8 ± 1.7	2.00 ± 0.15
*g*	*−3.01 (−4.13*–*−1.89)*	0.70 (−0.09–1.50)	-	*−4.12 (−5.47*–*−2.76)*	*−0.84 (−1.64*–*−0.04)*
40	22.1 ± 1.7	0.32 ± 0.06	45.1 ± 4.5	24.7 ± 1.5	1.82 ± 0.13
*g*	*−2.24 (−3.22*–*−1.26)*	*1.19 (0.36–2.03)*	−0.11 (−0.88–0.66)	*−3.10 (−4.24*–*−1.96)*	*−1.84 (−2.75*–*−0.92)*
60	23.3 ± 1.6	0.37 ± 0.07	44.2 ± 4.0	26.6 ± 1.4	1.66 ± 0.10
*g*	*−1.62 (−2.51*–*−0.74)*	*1.74 (0.84–2.65)*	−0.32 (−1.09–0.46)	*−2.11 (−3.07*–*−1.15)*	*−2.82 (−3.91*–*−1.74)*
80	25.7 ± 2.0	0.38 ± 0.08	43.3 ± 4.2	28.5 ± 1.5	1.52 ± 0.11
*g*	−0.35 (−1.13–0.42)	*1.70 (0.80–2.59)*	−0.52 (−1.31–0.26)	*−0.89 (−1.70*–*−0.09)*	*−3.62 (−4.87*–*−2.37)*
100	26.4 ± 1.9	0.42 ± 0.09	40.8 ± 4.1	29.9 ± 1.7	1.36 ± 0.09
*g*	-	*2.09 (1.13–3.05)*	*−1.07 (−1.90*–*−0.25)*	-	*−4.62 (−6.09*–*−3.15)*
Hexagonal Barbell Jump					
0	19.6 ± 1.9	0.26 ± 0.04	47.7 ± 6.0	21.2 ± 1.9	2.25 ± 0.20
*g*	*−4.05 (−5.40*–*−2.71)*	-	−0.14 (−0.91–0.63)	*−4.94 (−6.49*–*−3.40)*	-
20	21.3 ± 1.9	0.28 ± 0.05	48.5 ± 5.0	23.7 ± 1.8	2.05 ± 0.13
*g*	*−2.93 (−4.03*–*−1.82)*	0.45 (−0.33–1.23)	-	*−3.65 (−4.90*–*−2.39)*	*−1.16 (−1.99*–*−0.33)*
40	22.6 ± 2.1	0.32 ± 0.06	48.0 ± 5.2	25.2 ± 2.0	1.91 ± 0.12
*g*	*−2.07 (−3.02*–*−1.11)*	*1.12 (0.29–1.95)*	−0.10 (−0.87–0.67)	*−2.59 (−3.63*–*−1.55)*	*−2.06 (−3.01*–*−1.11)*
60	24.0 ± 2.1	0.35 ± 0.06	47.3 ± 5.5	26.8 ± 1.9	1.76 ± 0.12
*g*	*−1.30 (−2.15*–*−0.46)*	*1.61 (0.72–2.49)*	−0.23 (−1.00–0.55)	*−1.73 (−2.63*–*−0.83)*	*−2.90 (−4.00*–*−1.80)*
80	24.9 ± 1.5	0.39 ± 0.06	45.1 ± 5.5	28.1 ± 1.6	1.60 ± 0.12
*g*	*−0.95 (−1.76*–*−0.14)*	*2.33 (1.34–3.33)*	−0.63 (−1.42–0.16)	*−1.08 (−1.90*–*−0.25)*	*−3.85 (−5.14*–*−2.55)*
100	26.3 ± 1.3	0.41 ± 0.06	42.6 ± 4.2	29.8 ± 1.5	1.43 ± 0.10
*g*	-	*2.63 (1.58–3.68)*	*−1.25 (−2.10–−0.41)*	-	*−5.09 (−6.68*–*−3.51)*
Jump Shrug					
0	20.9 ± 1.8	0.23 ± 0.05	49.5 ± 6.7	23.8 ± 1.2	2.08 ± 0.22
*g*	*−2.81 (−3.89*–*−1.73)*	-	-	*−4.91 (−6.45*–*−3.37)*	-
20	21.8 ± 1.4	0.27 ± 0.07	48.8 ± 4.3	26.0 ± 1.2	1.88 ± 0.15
*g*	*−2.65 (−3.71*–*−1.60)*	0.58 (−0.21–1.36)	−0.13 (−0.90–0.64)	*−3.27 (−4.45*–*−2.10)*	*−1.04 (−1.86*–*−0.22)*
40	22.7 ± 1.2	0.30 ± 0.06	48.3 ± 4.2	27.5 ± 1.4	1.75 ± 0.12
*g*	*−2.23 (−3.21*–*−1.25)*	*1.20 (0.36–2.03)*	−0.22 (−0.99–0.56)	*−2.03 (−2.98*–*−1.09)*	*−1.76 (−2.66*–*−0.85)*
60	24.2 ± 1.0	0.33 ± 0.07	45.5 ± 4.5	28.7 ± 1.6	1.58 ± 0.10
*g*	*−1.25 (−2.09*–*−0.41)*	*1.58 (0.70–2.47)*	−0.69 (−1.48–0.10)	*−1.16 (−1.99*–*−0.33)*	*−2.80 (−3.88*–*−1.72)*
80	24.7 ± 1.3	0.37 ± 0.07	42.1 ± 5.1	29.5 ± 1.5	1.42 ± 0.13
*g*	*−0.82 (−1.62*–*−0.02)*	*2.40 (1.39–3.40)*	*−1.25 (−2.04*–*−0.37)*	−0.68 (−1.47–0.11)	*−3.48 (−4.69*–*−2.26)*
100	25.9 ± 1.5	0.40 ± 0.07	39.5 ± 4.1	30.6 ± 1.5	1.29 ± 0.10
*g*	-	*2.62 (1.57–3.67)*	*−1.75 (−2.65*–*−0.84)*	-	*−4.45 (−5.89*–*−3.02)*

*Notes*: MF = mean force; PP = peak power output; F_PP_ = force at PP; V_PP_ = velocity at PP; Hedge’s g effect sizes indicate the difference in relation to the greatest magnitude or shortest duration produced for each variable. *Italics* = indicates that the effect sizes are practically meaningful.

## Data Availability

The data presented within the current study may be available upon request; however, these data are not displayed in a public archive.
